# Wild-Type Anaplastic Lymphoma Kinase Expression in Solitary Pulmonary Nodules: A Potential Marker for Primary Lung Squamous Cell Carcinoma in Patients With Previous Neck Squamous Cell Carcinoma

**DOI:** 10.7759/cureus.58051

**Published:** 2024-04-11

**Authors:** Yusuke Takanashi, Kazuya Shinmura, Keigo Sekihara, Rei Ishikawa, Kazuhito Funai

**Affiliations:** 1 First Department of Surgery, Hamamatsu University School of Medicine, Hamamatsu, JPN; 2 Department of Tumor Pathology, Hamamatsu University School of Medicine, Hamamatsu, JPN

**Keywords:** immunohistochemistry, anaplastic lymphoma kinase, lung squamous cell carcinoma, laryngeal cancer, head and neck squamous cell carcinoma

## Abstract

In patients with a history of head and neck squamous cell carcinoma (HNSCC), distinguishing between primary lung squamous cell carcinoma (LSCC) and pulmonary metastasis of HNSCC is critical when a solitary pulmonary nodule is observed. However, differentiation in clinical practice remains challenging because no golden-standard immunohistochemical (IHC) marker has been established to identify the primary organ of squamous cell carcinoma (SCC). The anaplastic lymphoma kinase (*ALK*) gene harbors rearrangements in approximately 4-6% of non-small cell lung cancer (NSCLC) cases. The detection of *ALK* rearrangements is well-established through anti-ALK IHC. While anti-ALK IHC is primarily positive in adenocarcinoma within NSCLC, wild-type ALK without rearrangements is occasionally detected in other histological types, such as SCC. We report two surgical cases with a history of laryngeal cancer that exhibited solitary pulmonary SCC, in which only the lung lesions demonstrated positivity for wild-type ALK through IHC and fluorescence in-situ hybridization method, allowing for the diagnosis of primary LSCC and following postoperative strategy.

## Introduction

In patients with a history of head and neck squamous cell carcinoma (HNSCC), distinguishing between primary lung squamous cell carcinoma (LSCC) and pulmonary metastasis of HNSCC is critical when a solitary pulmonary nodule is observed [1]. This distinction is vital due to the significantly different treatment approaches for each disease. However, differentiation in clinical practice remains challenging. This difficulty arises because both diseases share similar patient backgrounds (with smoking as a risk factor), and there is no golden-standard immunohistochemical (IHC) marker to identify the primary origin of squamous cell carcinoma (SCC) [1]. Consequently, there is a need to establish new differential markers.

The anaplastic lymphoma kinase (ALK) gene is known to undergo rearrangements in approximately 4-6% of non-small cell lung cancer (NSCLC) cases [2,3]. These rearrangements amplify expression, often forming fusion genes with other genes, such as echinoderm microtubule-associated protein like-4 [2,4] and kinesin family member 5B [3]. The detection of ALK rearrangements is well-established through anti-ALK IHC and is used to determine the application of ALK-inhibitor therapy in NSCLC treatment [5-7]. While anti-ALK IHC is primarily positive in adenocarcinoma (ADC) within NSCLC, wild-type ALK without rearrangements is occasionally detected in other histological types (e.g., squamous cell, large-cell neuroendocrine, and small-cell carcinomas) [8].

We report two surgical cases with a history of laryngeal cancer that exhibited solitary pulmonary SCC, in which only the lung lesions demonstrated positivity for wild-type ALK through IHC and fluorescence in-situ hybridization (FISH) method, allowing for the diagnosis of primary LSCC.

## Case presentation

Case 1

**Figure 1:**
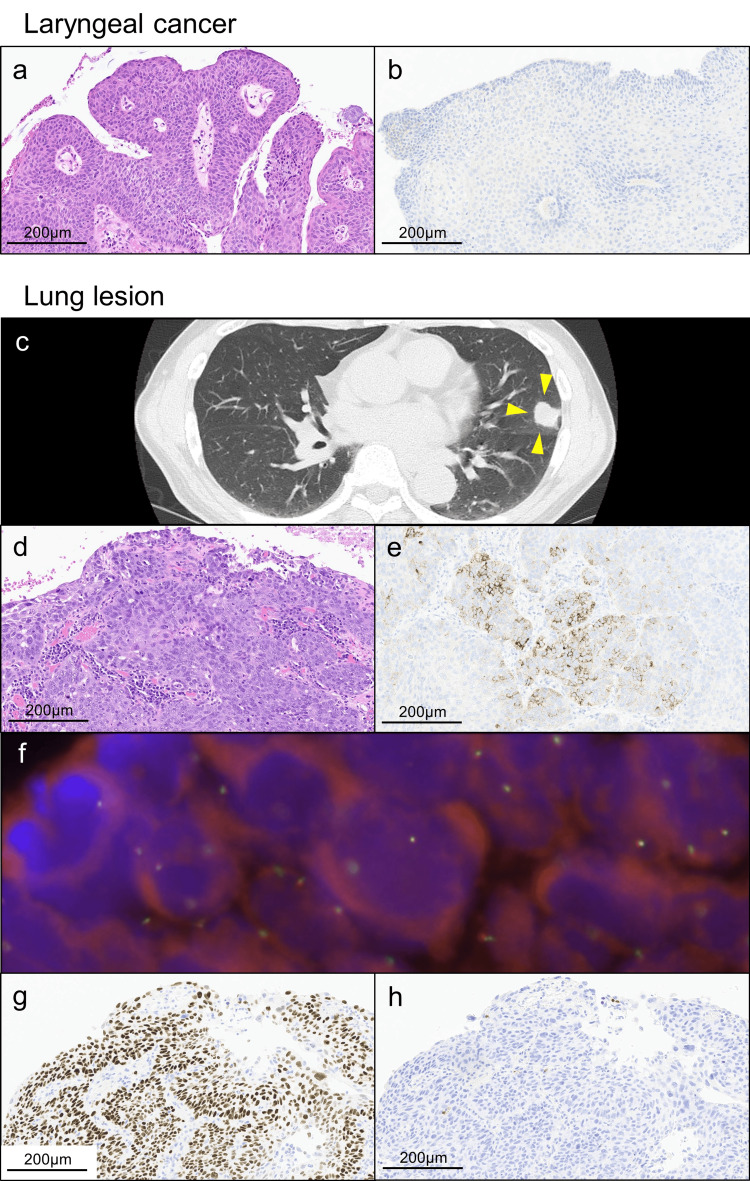
Radiological image and histopathological examinations. Histopathological examination of the left vocal cord tumor demonstrated well-differentiated squamous cell carcinoma (SCC) forming papillary proliferation (a) and negative immunohistochemistry (IHC) of anaplastic lymphoma kinase (ALK) (b). Eight months after the radiation therapy for laryngeal cancer, computed tomography (CT) showed a nodule, 2.2 × 2.0 cm, in the left upper lobe (c) (arrowheads). Histopathological examination of the lung lesion demonstrated moderately differentiated SCC (d) with heterogeneous staining of ALK IHC (e). However, ALK break-apart fluorescence in-situ hybridization (FISH) demonstrated no rearrangements (f). Other IHC findings included p40 positive (g) and thyroid transcription factor-1/napsin A negative (h), supporting pure SCC.

A 62-year-old male, an ex-smoker with a 47-pack-year smoking history, was diagnosed with laryngeal cancer (cT1aN0M0 stage I, Union for International Cancer Control (UICC) 8th edition); the biopsy of the left vocal cord demonstrated well-differentiated SCC forming papillary proliferation (Figure 1a). The patient received radiation therapy (RT) (66Gy), and the complete remission was achieved. Eight months after the RT, follow-up computed tomography (CT) revealed a nodule, 2.2 × 2.0 cm in diameter, in the S4 segment of the left upper lobe (Figure 1c). No significant lymphadenopathy was seen. The laryngeal fiberscope detected no local recurrence of the laryngeal cancer. Radiological workups, including positron emission tomography (PET) and brain magnetic resonance imaging (MRI) for further distant metastasis search, could not be performed due to claustrophobia. No elevation was observed in several tumor markers suggestive of primary lung or recurrent laryngeal cancer. Based on these examinations, the lung lesion was considered to be either primary lung cancer (cT1bN0M0 stage IA2, UICC 8th edition) or pulmonary metastasis of laryngeal cancer. As we anticipated difficulty in securing sufficient resection margins with a partial resection, we performed robotic-assisted left upper lobectomy with lymph node dissection.

Histopathological examination of the lung lesion demonstrated moderately differentiated SCC (Figure 1d). Notably, ALK (D5F3) (product code: 518-113735, Ventana Medical Systems, Inc., Tucson, AZ, USA) was focally positive, showing a heterogeneous staining pattern, on the lung lesion (Figure 1e), which prompted us to perform ALK break-apart FISH (product code: J17917, Jokoh Co. Ltd., Tokyo, Japan) to demonstrate no ALK rearrangements (Figure 1f). Other IHC findings included p40-positive (product code: 718171, Nichirei Biosciences Inc., Tokyo, Japan) (Figure 1g) and thyroid transcription factor-1 (TTF-1) (clone: SPT24, Novocastra, San Ramon, CA, USA)/napsin A (clone: IP64, Novocastra, San Ramon, CA, USA)-negative (Fig. 1h), supported pure SCC. Following the ALK positivity of the lung lesion, we retrospectively performed ALK IHC on the laryngeal cancer specimen for differential diagnosis and demonstrated ALK negativity (Figure 1b). Based on the difference in the ALK expression between laryngeal cancer and the lung lesion, we diagnosed the lung lesion as primary lung SCC (pT1bN0M0 stage IA2). The patient has been progression-free for 11 months following surgery.

Case 2

**Figure 2:**
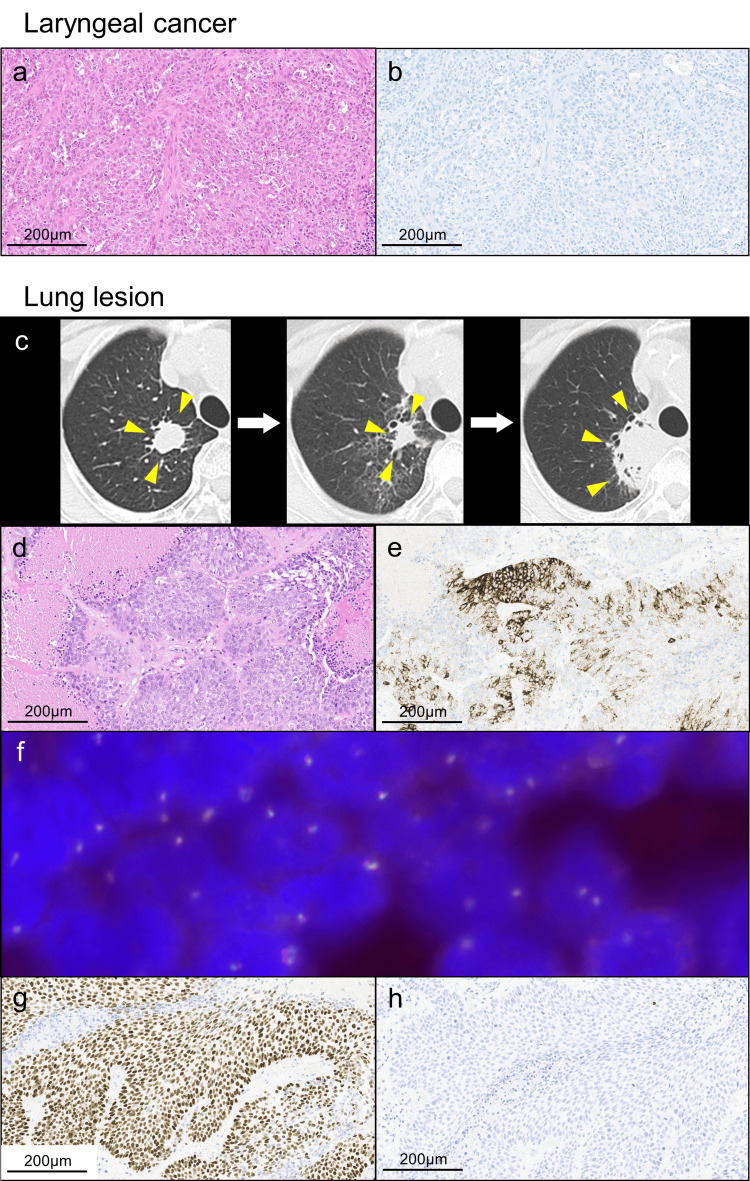
Radiological images and histopathological examinations. Histopathological examination of laryngeal cancer demonstrated moderately differentiated squamous cell carcinoma (SCC) (a) and negative immunohistochemistry (IHC) of anaplastic lymphoma kinase (ALK) (b). Eleven years after the chemoradiotherapy for laryngeal cancer, computed tomography (CT) showed a nodule, 1.8 × 1.7, in the right upper lobe (c) (left panel). Although radiation therapy resulted in a reduction of the lung lesion (1.4 × 1.4 cm) (c) (central panel), an increase of the lung lesion (4.3 × 2.4 cm) was observed seven months later (c) (right panel). Histopathological examination of the lung lesion demonstrated poorly differentiated SCC (d) with heterogeneous staining of ALK IHC (e). In this case, as well, ALK break-apart fluorescence in-situ hybridization revealed no rearrangements (f). Other IHC findings included p40 positive (g) and thyroid transcription factor-1/napsin A negative (h), demonstrating pure SCC.

A 76-year-old female, an ex-smoker with a 25-pack-year smoking history, received a total laryngectomy with bilateral neck dissection for laryngeal cancer (pT3N2bM0 stage IVA, UICC 8th edition). Histopathological examination demonstrated moderately differentiated SCC (Figure 2a) with right-mid and upper deep cervical lymph node metastases, and the patient received adjuvant chemoradiotherapy (bolus-cisplatin (80 mg/m^2^) × 3 courses, RT 66Gy). Eleven years after the surgery, a follow-up CT detected a mass, 1.8 × 1.7 cm in diameter, in the right upper lobe (Figure 2c, left panel). Transbronchial biopsy of the lung lesion demonstrated SCC. PET and brain MRI did not detect other metastatic lesions nor local recurrence of the laryngeal cancer. Thus, the lung lesion was considered to be either primary lung SCC (cT1bN0M0 stage IA2, UICC 8th edition) or pulmonary metastasis of the laryngeal cancer. We administered RT (60Gy) for a lung lesion, which resulted in a reduction of the lesion (1.4×1.4 cm) (Fig. 2c, central panel). However, seven months later, an enlargement of the lung lesion (4.3×2.4 cm) with suspected mediastinal invasion was observed, leading to a diagnosis of local recurrence (Fig. 2c, right panel). Under the diagnoses of primary lung SCC (ycT4N0M0 Stage IIIA, UICC 8th edition) or pulmonary metastasis of laryngeal cancer, we performed a right upper lobectomy with lymph node dissection.

Histopathological examination of the lung lesion demonstrated poorly differentiated SCC associated with necrosis and viable tumor cells (Figure 2d). ALK was focally positive, showing a heterogeneous staining pattern (Figure 2e). However, ALK break-apart FISH did not detect ALK rearrangement (Figure 2f). IHC findings included p40 positive (Figure 2g) and TTF-1/napsin A negative (Figure 2h), supporting pure SCC. In addition, metastasis to the mediastinal lymph nodes was observed. In this case, as well, we retrospectively performed ALK IHC on the laryngeal cancer specimen and proved ALK negativity (Figure 2b). Based on the difference in ALK expression between the laryngeal and the lung SCC, we diagnosed the lung lesion as primary lung SCC (ypT1cN2M0 stage IIIA). We administered four courses of adjuvant chemotherapy (split-cisplatin (80 mg/m^2^) + gemcitabine (1000 mg/m^2^)), and the patient has been progression-free for nine months following surgery.

## Discussion

Methods for distinguishing pulmonary metastasis of HNSCC from primary LSCC are generally categorized into three types: diagnostic approaches using clinical parameters, pathological methods including hematoxylin and eosin (H&E) staining and IHC findings, and molecular analyses.

Proposed clinical parameters suggesting primary LSCC include solitary lung lesions located centrally in the lung parenchyma, a disease-free interval longer than three to five years, and early-stage HNSCC with no locoregional recurrence [1]; however, none are definitive for diagnosis. In view of pathological methods, HNSCC and primary LSCC often differ in their histological grades - HNSCC commonly presents as well-differentiated with keratinization, while primary LSCC tends to be non-keratinized and of moderate to low differentiation [1]. However, since the grade of differentiation can vary between primary tumors and their metastases, this is not a conclusive finding. There is a subgroup of oropharyngeal cancers caused by human papillomavirus (HPV) infection, which has recently been on the rise [9]. The IHC for p16 is reported to be a potentially useful tool in the differential diagnosis of solitary pulmonary nodules with a history of HPV-related oropharyngeal cancer; however, its utility in other types of HNSCC is not reported [1]. Ichinose et al. have reported an excellent discriminatory ability (90-96% sensitivity, 44-62% specificity, and 65-77% accuracy) in distinguishing HNSCC from LSCC using an IHC diagnostic panel for three antigens (cytokeratin 19, matrix metalloproteinase-3, and phosphoinositide 3-kinase) [10]; although this protocol is well-developed, it has not yet been widely implemented in clinical practice, possibly because the antibodies used are not routinely employed in the diagnosis of HNSCC or LSCC. In molecular analysis, several gene panels have been proposed, but their clinical application is limited due to the complexity and cost involved [11,12].

In detecting ALK rearrangement in NSCLC, the results of highly-sensitive ALK IHC correlate well with those of FISH analysis. Consequently, simple ALK IHC has been established as a reliable method in routine clinical practice [5-7]. On the other hand, expression of wild-type ALK without rearrangement is detected as a heterogeneous staining pattern by ALK IHC [8]. In the two cases we reported, ALK IHC showed a heterogeneous staining pattern, and ALK break-apart FISH was negative, suggesting the detection of wild-type ALK expression. In addition, the adenocarcinoma markers, TTF-1 and napsin A, were negative across the entire tumor cross-section, leading us to rule out the possibility of ALK expression in the adenocarcinoma component of SCC.

In our reported case 1, the diagnosis of primary LSCC was supported by the early stage of laryngeal cancer, the solitary nature of the lung lesion, and differing grades of differentiation between the laryngeal cancer (well-differentiated) and the lung lesion (moderately differentiated). The definitive diagnosis was based on the ALK IHC positivity, exclusive to the lung lesion. As a postoperative strategy, continued follow-up observation for LSCC (pT1bN0M0 stage IA2) and laryngeal cancer was decided. In case 2, the solitary nature of the lung lesion, a long disease-free interval of 11 years, and differing grades of differentiation between the laryngeal cancer (moderately differentiated) and the lung lesion (poorly differentiated) supported the diagnosis of primary LSCC. However, as laryngeal cancer was an advanced stage (pT3N2bM0 stage IVA) with lymph node metastasis, a pulmonary metastasis recurrence of laryngeal cancer could not be completely ruled out. Similar to case 1, the diagnosis of LSCC was confirmed based on the ALK IHC positivity exclusive on the lung lesion; postoperative adjuvant chemotherapy was decided for the primary LSCC with lymph node metastasis, which likely improved the patient’s prognosis.

Because the frequency of wild-type ALK expression in HNSCC and primary LSCC is unknown, its efficacy in differential diagnosis may be limited. However, given the current scarcity of practical differentiation markers and the availability of ALK IHC for routine diagnosis in NSCLC, conducting a study to compare the positive rate of ALK IHC in HNSCC and LSCC cohorts could suggest the value of ALK IHC as a novel differentiation marker.

## Conclusions

We reported two cases in which wild-type ALK was detected in a solitary pulmonary SCC with a history of HNSCC using ALK IHC, and the pulmonary nodule was diagnosed as primary LSCC. Reaching a definitive diagnosis for the pulmonary nodules led to the determination of appropriate postoperative treatment strategies. While the efficacy of ALK IHC in differentiating pulmonary metastasis of HNSCC from primary LSCC may be limited, considering the current scarcity of practical differentiation markers and the excellent accessibility to utilize ALK IHC in routine clinical practice, its potential as a novel differentiation marker warrants further study and validation.
